# DcMYB113, a root‐specific R2R3‐MYB, conditions anthocyanin biosynthesis and modification in carrot

**DOI:** 10.1111/pbi.13325

**Published:** 2020-01-22

**Authors:** Zhi‐Sheng Xu, Qing‐Qing Yang, Kai Feng, Xiao Yu, Ai‐Sheng Xiong

**Affiliations:** ^1^ State Key Laboratory of Crop Genetics and Germplasm Enhancement Ministry of Agriculture and Rural Affairs Key Laboratory of Biology and Germplasm Enhancement of Horticultural Crops in East China College of Horticulture Nanjing Agricultural University Nanjing China

**Keywords:** DcMYB113, anthocyanin, root‐specific, modification, WGCNA, carrot

## Abstract

Purple carrots, the original domesticated carrots, accumulate highly glycosylated and acylated anthocyanins in root and/or petiole. Previously, a quantitative trait locus (QTL) for root‐specific anthocyanin pigmentation was genetically mapped to chromosome 3 of carrot. In this study, an *R2R3‐MYB* gene, namely *DcMYB113*, was identified within this QTL region. *DcMYB113* expressed in the root of ‘Purple haze’, a carrot cultivar with purple root and nonpurple petiole, but not in the roots of two carrot cultivars with a purple root and petiole (Deep purple and Cosmic purple) and orange carrot ‘Kurodagosun’, which appeared to be caused by variation in the promoter region. The function of *DcMYB113* from ‘Purple haze’ was verified by transformation in ‘Cosmic purple’ and ‘Kurodagosun’, resulting in anthocyanin biosynthesis. Transgenic ‘Kurodagosun’ carrying *DcMYB113* driven by the CaMV 35S promoter had a purple root and petiole, while transgenic ‘Kurodagosun’ expressing *DcMYB113* driven by its own promoter had a purple root and nonpurple petiole, suggesting that root‐specific expression of *DcMYB113* was determined by its promoter. DcMYB113 could activate the expression of *DcbHLH3* and structural genes related to anthocyanin biosynthesis. *DcUCGXT1* and *DcSAT1*, which were confirmed to be responsible for anthocyanins glycosylation and acylation, respectively, were also activated by DcMYB113. The WGCNA identified several genes co‐expressed with anthocyanin biosynthesis and the results indicated that *DcMYB113* may regulate anthocyanin transport. Our findings provide insight into the molecular mechanism underlying root‐specific anthocyanin biosynthesis and further modification in carrot and even other root crops.

## Introduction

Carrots (*Daucus carota* L.; 2*n* = 2*x* = 18) are one of the most economically important vegetables in the world and contain various components with health‐promoting properties in humans. The original domesticated carrots were purple and yellow. There is documentation that these cultivars existed in Central Asia in approximately in the 10th century, and yellow carrot is thought to be a colour mutant of purple carrot (Banga, 1963). From this primary centre, purple‐ and yellow‐rooted carrots spread to Western and eastern countries. White carrots and red carrots were subsequently noted in Europe and China, respectively. In European countries, people preferred yellow carrots until orange carrots originated in the Netherlands in the 16th century and gradually supplanted carrots of other colours (Arscott and Tanumihardjo, 2010).

From the viewpoint of taproot colour, cultivated carrots can be mainly divided into five types: orange, yellow, red, white, and purple. The orange, yellow, and red pigmentation of carrots results from high quantities of carotene, lutein, and lycopene, respectively, while white carrots accumulate very low levels of carotenoids (Arscott and Tanumihardjo, 2010; Clotault *et al.*, 2008). The purple pigmentation of carrots is due to the presence of high levels of anthocyanins (Kammerer *et al.*, 2004; Montilla *et al.*, 2011).

The genetic factors controlling anthocyanin biosynthesis have been well studied in many plant species. The expression of structural genes encoding enzymes directly involved in anthocyanin synthesis is controlled by transcription factors (TFs). In many plant species, the expression of structural genes in the anthocyanin biosynthetic pathway is directly activated by the MBW complex, comprising R2R3‐MYB TFs, basic‐helix‐loop‐helix (bHLH) TFs and WD‐repeat proteins (Chagne *et al.*, 2013; Espley *et al.*, 2007; Jian *et al.*, 2019; Jin *et al.*, 2016; Peng *et al.*, 2019; Tian *et al.*, 2015). The R2R3‐MYB TFs were often identified as determinants of variation in anthocyanin pigmentation.

Nowadays, the distribution of purple pigmentation in root and petiole differs among purple carrot cultivars. The purple carrots with a purple petiole had a solid purple root (purple periderm, phloem, and xylem) or purple peridermal root (purple periderm but nonpurple phloem and xylem), while the purple carrots with a nonpurple petiole had a purple root with purple periderm and phloem but nonpurple xylem. In previous studies, two genes, *P_1_* and *P_3_*_,_ which condition anthocyanin pigmentation of carrot roots, were genetically mapped to chromosome 3 (Cavagnaro *et al.*, 2014). *P_1_* is an inherited dominant gene controlling anthocyanin biosynthesis in carrots with a purple root and nonpurple petiole. *P_3_*, a different gene from *P_1_*, is responsible for anthocyanin pigmentation in carrots with a purple root and petiole. Cavagnaro et al. suggested that independent mutations of the genes controlling carrot anthocyanin pigmentation within these two loci and human selection events may have contributed to the domestication of purple carrots (Cavagnaro *et al.*, 2014). As described in previous studies, the structural genes controlling anthocyanin biosynthesis in carrot have been identified and characterized (Xu *et al.*, 2014; Yildiz *et al.*, 2013). The expressions of all those structural genes were found to be positively correlated with anthocyanin accumulation in carrots, but no candidate for *P_1_* or *P_3_* was identified. Variation in the activity of regulatory genes is thought to account for the variable production of anthocyanins in carrot. In our previous report, we identified *DcMYB6*, an *R2R3‐MYB* gene involved in carrot root anthocyanin biosynthesis within the *P_3_* region (Xu *et al.*, 2017). Iorizzo et al. identified an *R2R3‐MYB* gene, *DcMYB7*, within the *P_3_* region as a candidate for the gene controlling anthocyanin biosynthesis in carrot with a purple root and petiole (Iorizzo *et al.*, 2019). Recently, we confirmed that *DcMYB7* is *P_3_* (Xu *et al.*, 2019b). However, the key gene within the *P_1_* region that controls anthocyanin biosynthesis in carrot with a purple root and nonpurple petiole is still unknown.

The anthocyanin composition in purple carrots has been studied in detail. Purple carrots contain anthocyanins derived from three anthocyanidin aglycones: cyanidin, peonidin and pelargonidin. The purple pigments in carrots are almost exclusively cyanidin‐based anthocyanins (Kammerer *et al.*, 2004; Montilla *et al.*, 2011). In various plants, anthocyanins are attached with different moieties to various positions, leading to the generation of hundred types of anthocyanins (Kong *et al.*, 2003). The genes responsible for sequential modification are different among various plant species. After anthocyanidins have been catalysed into relative stable glycosylated forms by DcUCGalT1, they further undergo a series of glycosylation and acylation reactions, which generate more stable modified anthocyanins (Cavagnaro *et al.*, 2014; Xu *et al.*, 2016). With the advantages of stability and health‐promoting properties, anthocyanins from purple carrots are widely used in various foods and are the third largest natural commercial food colorant (Butelli *et al.*, 2008; Netzel *et al.*, 2007). Several quantitative trait loci (QTL) related to anthocyanin modifications in carrot were proposed (Cavagnaro *et al.*, 2014). However, the genes responsible for further glycosylation and acylation of anthocyanins in carrot were not identified or characterized in their study. Recently, we found two genes encoding a glycosyltransferase and an acyltransferase, respectively, within two QTLs associated with the content of glycosylated and acylated anthocyanins (Xu *et al.*, 2019b). But their glycosyltransferase or acyltransferase activities were not determined.

In the present study, we identified an *R2R3‐MYB* gene, namely *DcMYB113*, within the *P_1_* locus. Gene expression and stable plant transformation analyses confirmed that *DcMYB113* was responsible for root‐specific anthocyanin pigmentation. We also found that *DcMYB113* could condition anthocyanins for further glycosylation and acylation by up‐regulating two genes encoding a glycosyltransferase and an acyltransferase, respectively. The results of our study not only provide evidence of the molecular mechanisms underlying differences in anthocyanin pigmentation among domesticated carrots, but also provide valuable data for breeding programs aiming to increase the amount and stability of anthocyanins in carrot and other plant species.

## Results

### Identification of *DcMYB113* gene

**Figure 1:**
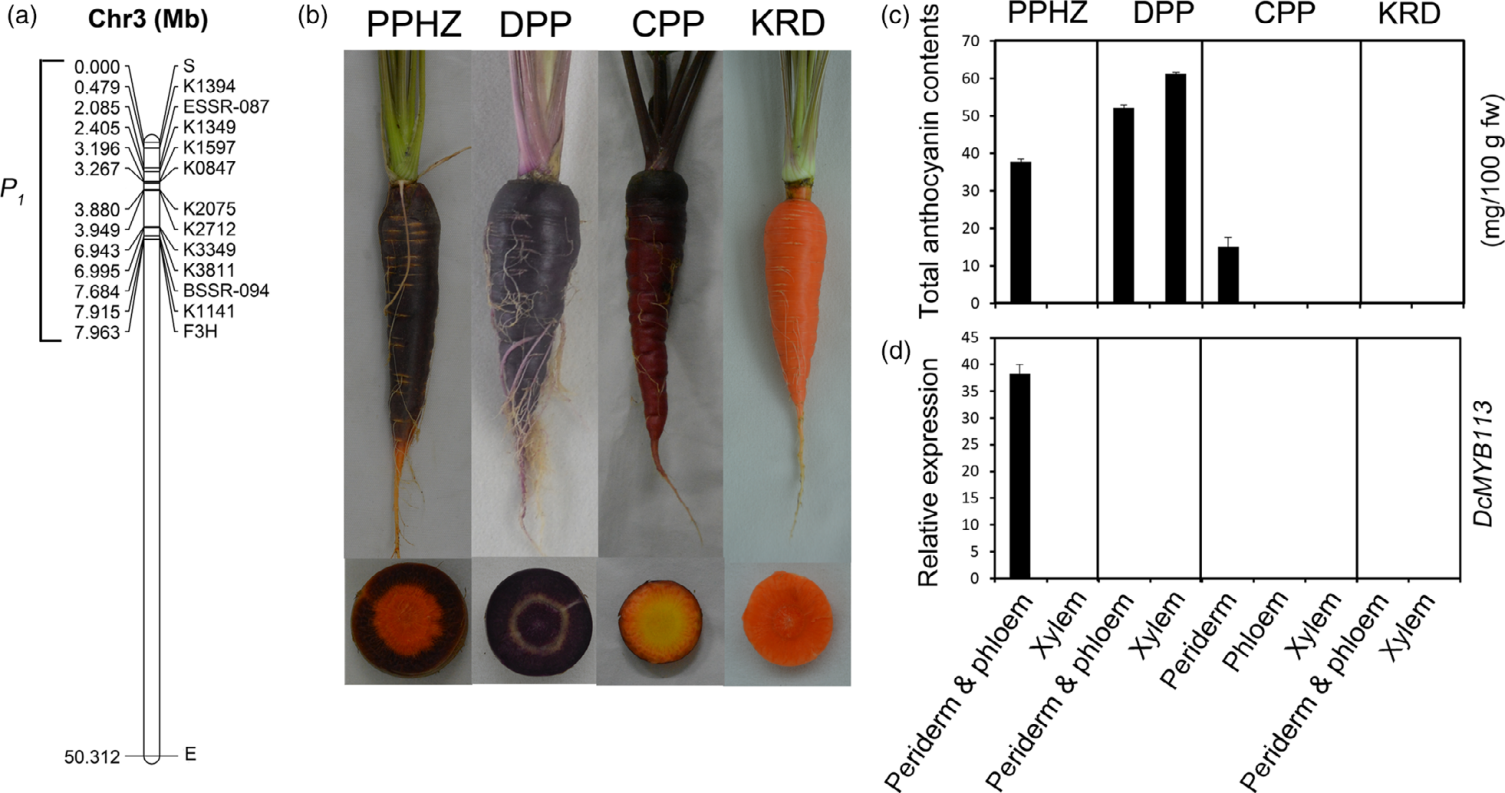
Identification of *DcMYB113* in carrot. (a) Site of *P_1_* locus on chromosome 3 of carrot. (b) Four different carrot cultivars used in this study at 90‐day‐old stage: PPHZ, ‘Purple haze’; DPP, ‘Deep purple’; CPP, ‘Cosmic purple’; and KRD, ‘Kurodagosun’. (c) Total anthocyanin contents in various root tissues of different carrot cultivars. Values are means of three independent experiments ± SDs. (d) Relative transcript levels of *DcMYB113* in different root tissues of various carrot cultivars. Data are means of biological triplicate qRT‐PCRs ± SD.

A previous study suggested that *P_1_*, a gene positively associated with anthocyanin accumulation in carrot with a purple root and nonpurple petiole, was genetically mapped within the region between SNP marker K1394 and the *DcF3H1* gene on chromosome 3 (Figure 1a) (Cavagnaro *et al.*, 2014). In total, 812 genes, from DCAR_008672 to DCAR_009483 (*DcF3H1*), were located in this region. To identify the candidate gene for *P_1_*, transcriptomes of roots at 3‐month‐old stage from ‘Purple haze’ (PPHZ), ‘Deep purple’ (DPP), ‘Cosmic purple’ (CPP) and ‘Kurodagosun’ (KRD) were sequenced (Figure 1b). The ‘periderm & phloem’ tissues of PPHZ root, all tissues of DPP root and periderm tissue of CPP root accumulated massive quantities of anthocyanins, while anthocyanins in the other root tissues were undetectable (Figure 1c).

In total, 748 genes were identified within the target region based on the transcriptomes of PPHZ, DPP, CPP and KRD (Table S1). The previous study reported that anthocyanin pigmentation in roots of DPP and CPP was controlled by *P_3_* (Cavagnaro *et al.*, 2014). Thus, comparative transcriptome analyses of RNA‐Seq data of purple root ‘periderm & phloem’ tissues of PPHZ *vs.* the other samples were conducted to identify differentially expressed genes within the *P_1_* region. Eleven genes were up‐regulated more than fivefold in the purple periderm and phloem tissues of PPHZ compared with other samples (Table S2). Three of them, DCAR_008994, DCAR_009022 and DCAR_009453, showed higher than 2 fragments per kilobase per total million mapped reads (FPKM) expression levels in the root ‘periderm & phloem’ tissues of PPHZ. The genes DCAR_008994, DCAR_009022 and DCAR_009453 encode an R2R3‐MYB protein, an AMP‐dependent synthetase/ligase family protein, and a U‐box domain‐containing protein kinase family protein, respectively. DCAR_008994 showed a much higher expression level (146.70 FPKM) than DCAR_009022 (3.46 FPKM) and DCAR_009453 (5.47 FPKM) in the purple periderm and phloem tissues of PPHZ. In a phylogenetic analysis of the DCAR_008994 and R2R3‐MYB families from *Arabidopsis*, DCAR_008994 and *AtMYB113* clustered into the same clade of proteins that were known to regulate the anthocyanin biosynthetic pathway (Figure S1). Thus, DCAR_008994 (hereafter, *DcMYB113*) was identified as a candidate gene for the control of anthocyanin biosynthesis in the purple ‘periderm & phloem’ tissues of PPHZ. qRT‐PCR analyses indicated that *DcMYB113* showed high transcript levels in the ‘periderm & phloem’ tissues of PPHZ root and undetectable levels in the xylem tissue of PPHZ root. *DcMYB113* expression was undetectable in all the root tissues of DPP, CPP, and KRD (Figure 1d).

### Overexpression of *DcMYB113* from PPHZ in KRD and CPP

**Figure 2:**
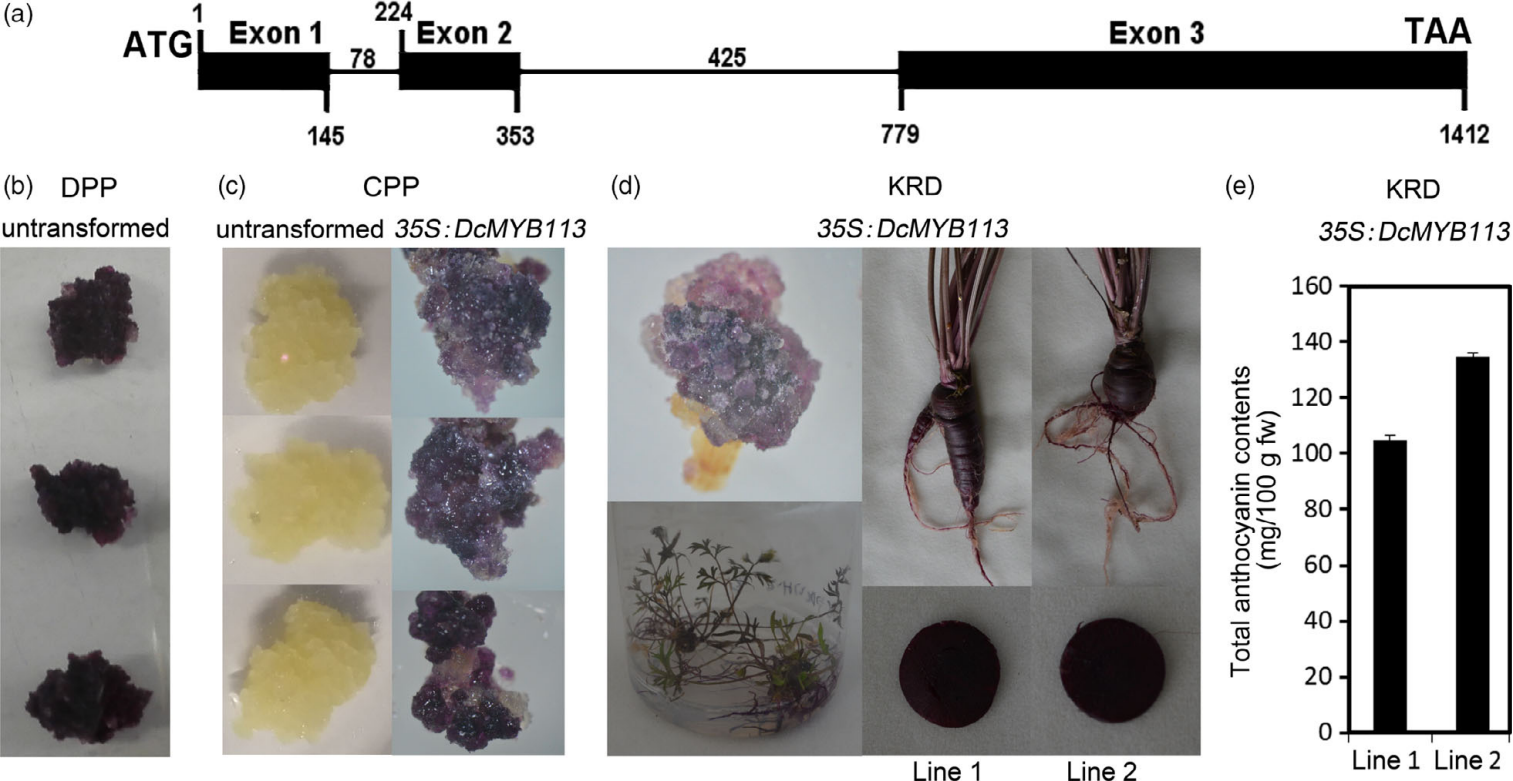
Functional test of *DcMYB113* in carrots. (a) Schematic representing *DcMYB113* from PPHZ with exons (black boxes) and introns (black lines). (b) Purple calli produced from DPP explants. (c) Calli generated from CPP explants without transformation or transformed with *35S:DcMYB113*. (d) Overexpression of *35S:DcMYB113* in KRD. (e) Total anthocyanin contents in roots of two *35S:DcMYB113* transgenic KRD lines. Values are means of three independent experiments ± SDs.

The cDNA and genomic DNA (gDNA) sequences of *DcMYB113* were cloned from PPHZ with DcMYB113‐F and DcMYB113‐R primers, which were designed according to the sequences of assembled RNA‐Seq reads from PPHZ root (Figure S2). The cDNA and gDNA sequences of *DcMYB113* were 909 and 1412 bp long, respectively (Figure S3), and contained an open reading frame (ORF) encoding 302 amino acids. The results of an alignment analysis showed that *DcMYB113* from PPHZ had three exons and two introns (Figure 2a). The three exons were 145, 120 and 634 bp long, and the two introns were 78 and 425 bp long, respectively.

DPP accumulated anthocyanins across the entire root section and their explants produced deep purple calli (Figure 2b). It is impossible to investigate the function of *DcMYB113* from PPHZ in DPP. Hence, *DcMYB113* from PPHZ was transformed into CPP and KRDs under the control of CaMV 35S promoter for functional test*.* CPP explants produced nonpurple calli without transformation and produced purple calli after transformed with *35S:DcMYB113* (Figure 2c). More than one thousand purple calli were produced from CPP explants after transformation; however, no plantlet was obtained because of the difficulty of regeneration. Purple calli were also produced from KRD explants after transformation and regenerated to produce purple plantlets (Figure 2d). After 90 days of growth, *35S:DcMYB113* KRD lines maintained a purple colour in the roots and petioles. The entire root cross section of different *35S:DcMYB113* KRD lines showed purple pigmentation, different from PPHZ roots having purple periderm and phloem tissues but nonpurple xylem tissue. The total anthocyanin contents in roots of 90‐day‐old plants were higher in the two *35S:DcMYB113* KRD lines than that in PPHZ (Figure 2e).

### Complementation of anthocyanin‐deficient phenotype of CPP and KRD with *DcMYB113* from PPHZ

**Figure 3:**
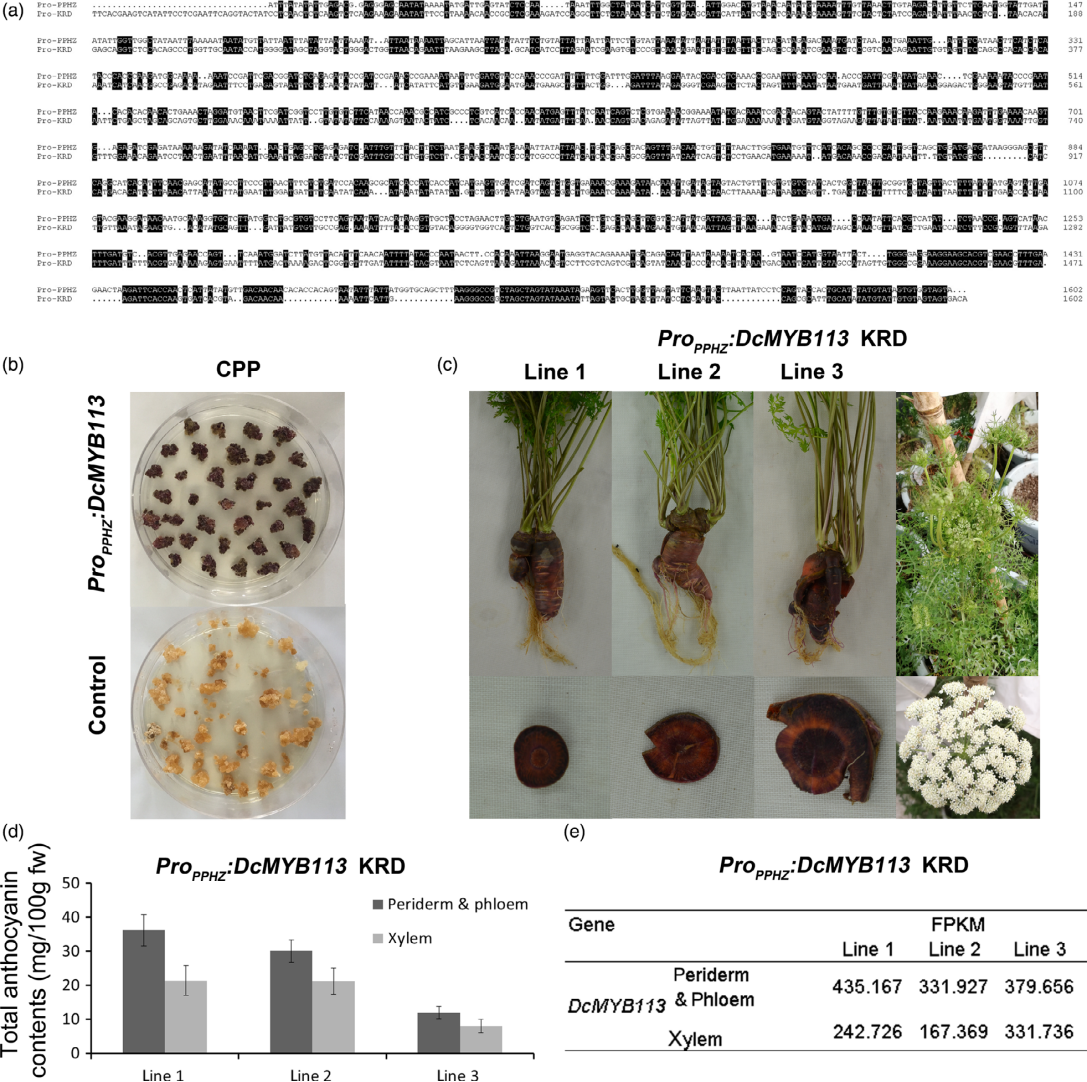
Complementation of CPP and KRD with *DcMYB113* from PPHZ. (a) Alignment analysis of promoter sequences of *DcMYB113* cloned from PPHZ (Pro‐PPHZ) and found in the KRD genome (Pro‐KRD). Identical sequences are shaded in black. (b) Calli generated from CPP explants transformed with *Pro_PPHZ_:DcMYB113* or pCAMBIA 1301 vector (as control). (c) The KRD lines expressing *Pro_PPHZ_:DcMYB113*. (d) Total anthocyanin contents in different root tissues of three *Pro_PPHZ_:DcMYB113* transgenic KRD lines. Data are means of three biological replicates ± SDs. (e) The expression level (FPKM) of *DcMYB113* in root tissues of three *Pro_PPHZ_:DcMYB113* transgenic KRD lines according to transcriptome data.

To investigate why *DcMYB113* showed high expression levels in the PPHZ root but undetectable levels in the KRD, CPP and DPP root, we tried to isolate its promoter sequence from the PPHZ. However, we could not obtain the promoter sequence using primers designed from sequences in the KRD or a doubled haploid carrot genomes (Iorizzo *et al.*, 2016; Wang *et al.*, 2018). Therefore, we assembled the raw reads (GenBank accession number: SRR2146943) of a purple carrot (GenBank accession number: SAMN03766331) with the same purple pigmentation phenotype as that of PPHZ. A scaffold containing the partial DNA sequence of *DcMYB113* was identified (Figure S4). Based on the sequences of this scaffold, the 2388‐bp promoter region of *DcMYB113* was amplified from gDNA of PPHZ with Pro_DcMYB113_‐F and Pro_DcMYB113_‐R primers (Table S3). However, the promoter region of *DcMYB113* was not able to be amplified from the gDNA of DPP, CPP and KRD with Pro_DcMYB113_‐F and Pro_DcMYB113_‐R primers, indicating the promoter region of *DcMYB113* from PPHZ was different from that from DPP, CPP and KRD. In an alignment, the promoter sequence of *DcMYB113* from PPHZ showed a very low degree of similarity to that from KRD genome (Figure 3a).

The promoter region and CDS region of *DcMYB113* was cloned from PPHZ to prepare the *Pro_PPHZ_:DcMYB113* construct. This construct was then genetically transformed into CPP and KRD. Expression of *Pro_PPHZ_:DcMYB113* in CPP explants led to the generation of purple calli, while CPP explants transformed with pCAMBIA 1301 vector produced nonpurple calli (Figures 3b). No plantlet was obtained because of the difficulty of regeneration. *DcMYB113* from PPHZ complemented the loss of function of *DcMYB113* in KRD root. Transgenic KRD with a purple root and nonpurple petiole were generated after transformed with *Pro_PPHZ_:DcMYB113* (Figure 3c). However, the roots of three *Pro_PPHZ_:DcMYB113* transgenic KRD lines had purple periderm, phloem and xylem tissues, different from PPHZ roots with purple periderm and phloem but nonpurple (orange) xylem tissues. After bolting, *Pro_PPHZ_:DcMYB113* transgenic KRD generated nonpurple pigmented inflorescences (Figure 3c).

The anthocyanin contents were determined in root tissues of the three *Pro_PPHZ_:DcMYB113* KRD lines. All three *Pro_PPHZ_:DcMYB113* transgenic KRD lines accumulated higher anthocyanin contents in the root ‘periderm & phloem’ tissues than that in xylem tissue (Figure 3d). Transcriptome data revealed that *DcMYB113* expressed in both ‘periderm & phloem’ and xylem tissues of *Pro_PPHZ_:DcMYB113* transgenic KRD roots and showed relative higher transcript levels in the ‘periderm & phloem’ tissues than that in xylem tissue (Figure 3e).

### Activation of *DcbHLH3* and anthocyanin biosynthetic genes by DcMYB113

**Figure 4:**
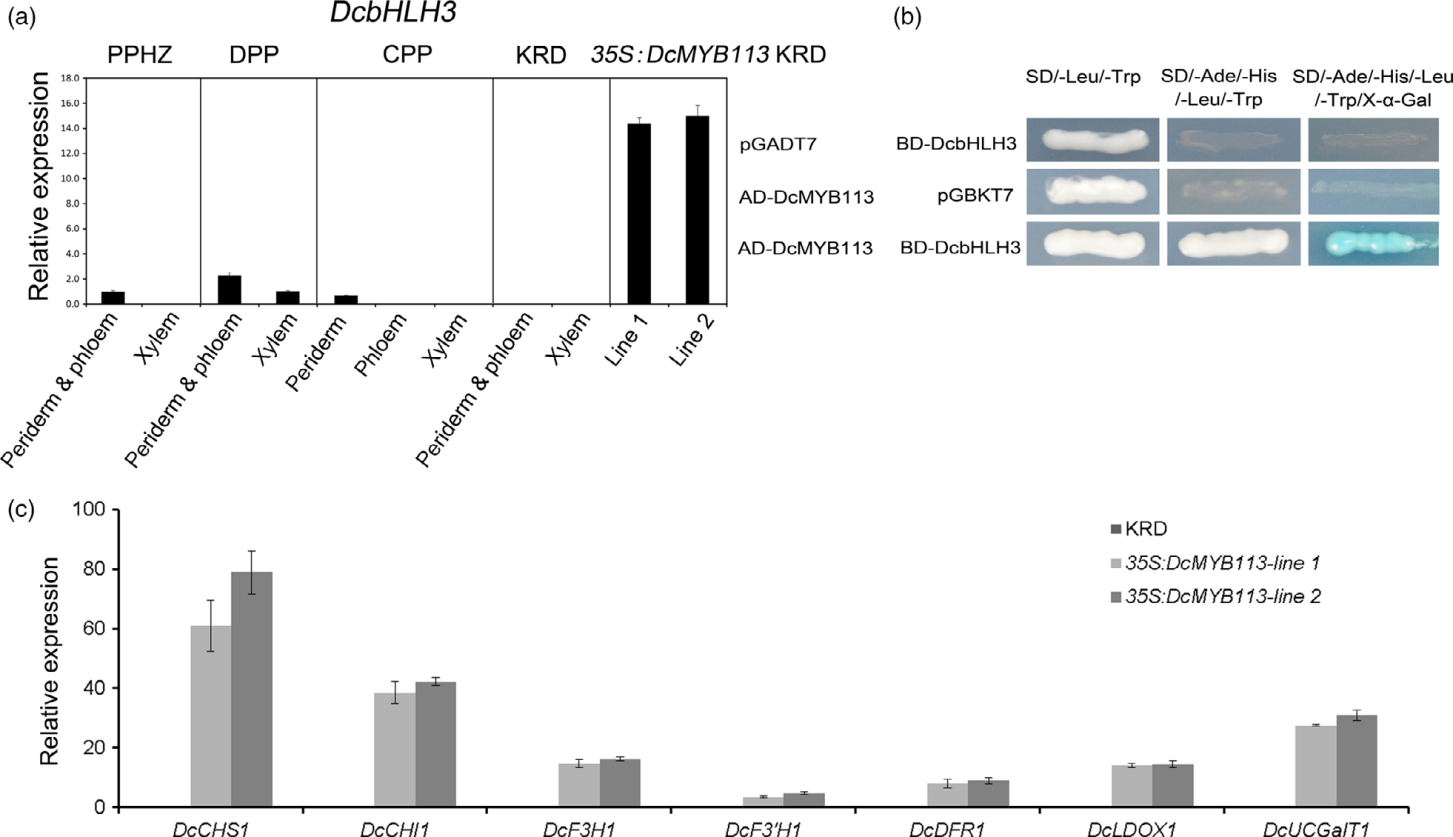
The role of *DcMYB113* on the expression of *DcbHLH3* and structural genes related to anthocyanin biosynthesis. (a) Relative transcript levels of *DcbHLH3* in different root tissues of various carrot cultivars and two *35S:DcMYB113* transgenic KRD lines. Data are means of biological triplicate qRT‐PCR ± SD. (b) Interaction between DcMYB113 with DcbHLH3 determined by yeast two‐hybrid assays. (c) Relative transcript levels of anthocyanin pathway structural genes in 90‐day‐old old roots of untransformed KRD and two *35S:DcMYB113* transgenic KRD lines. Data represents means of three biological replicates ± SDs.

In many plant species, MYBs interact with partner bHLHs to co‐regulate anthocyanin biosynthesis. In previous studies, MYBs were found to up‐regulate their bHLH interactors in *Arabidopsis* but not in apple (Espley *et al.*, 2007; Tohge *et al.*, 2005). A gene encoding a bHLH protein, *DcbHLH3* (GenBank accession number: MK572822), was identified by reference to its ortholog *MdbHLH3*, which has been shown to enhance anthocyanin biosynthesis in apple (Espley *et al.*, 2007). We found that *DcbHLH3* expression was associated with anthocyanin production, with *DcbHLH3* showing high transcript levels in all the purple root tissues of PPHZ, DPP and CPP roots, but undetectable transcript levels in all the nonpurple root tissues of PPHZ, CPP and KRD (Figure 4a). *DcbHLH3* showed greatly increased transcript levels in the two *35S:DcMYB113* transgenic KRD lines compared with the untransformed KRD (Figure 4a). In yeast two‐hybrid assays, DcMYB113 was capable of interacting with DcbHLH3 (Figure 4b). Previous studies have revealed that cyanidin‐based anthocyanins are almost exclusively responsible for the purple colour of carrots (Cavagnaro *et al.*, 2014; Kammerer *et al.*, 2004; Montilla *et al.*, 2011). Transcriptional analysis of structural genes involved in cyanidin‐based anthocyanins biosynthesis (*DcCHS1*, *DcCHI1*, *DcF3H1*, *DcF3'H1*, *DcDFR1*, *DcLDOX1* and *DcUCGalT1*) by qRT‐PCR indicated that all these genes were significantly up‐regulated in the two *35S:DcMYB113* transgenic KRD lines compared with the untransformed KRD (Figure 4c). Together, these results suggested that *DcMYB113* positively regulated the expression of *DcbHLH3* and all the tested structural genes in the anthocyanin biosynthesis pathway in carrot.

### Anthocyanin composition profile in transgenic KRD

**Figure 5:**
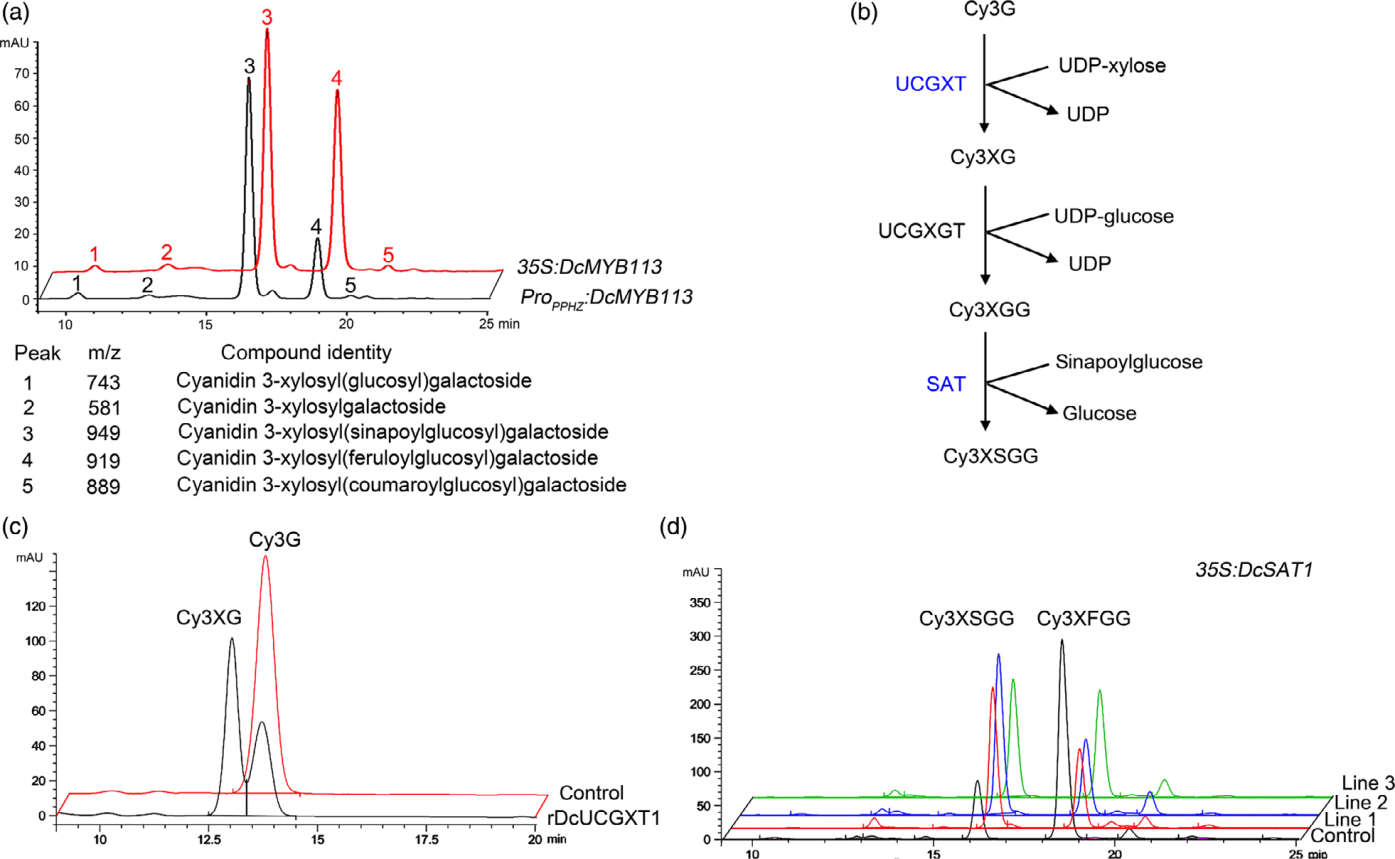
Role of *DcMYB113* in anthocyanin glycosylation and acylation. (a) Anthocyanin composition profile in *35S:DcMYB113* (line 1) and *Pro_PPHZ_:DcMYB113* (line 1) transgenic KRD roots. (b) Proposed enzymes participating in Cy3XSGG biosynthesis. Enzymes identified in this study are marked in blue. (c) HPLC‐MS analysis of products after incubation of cyanidin‐3‐*O*‐galactoside and UDP‐xylose with rDcUCGXT1 or crude protein (control) extracted from *E. coli* transformed with pET‐30a vector. (d) Anthocyanin composition profile in DPP calli carrying *35S:DcSAT1* or pCAMBIA 1301 vector (control).

In purple carrots, anthocyanins further undergo a series of glycosylation and acylation steps that make them more stable. The anthocyanin composition in different purple carrot cultivars has been analysed in several studies (Cavagnaro *et al.*, 2014; Kammerer *et al.*, 2004; Montilla *et al.*, 2011). In this study, the anthocyanin composition in roots of *35S:DcMYB113* (line 1) and *Pro_PPHZ_:DcMYB113* transgenic KRD (line 1) was determined by high‐performance liquid chromatography–mass spectrometry (HPLC‐MS). Five peaks were identified in the chromatograms of root extracts (Figure 5a). The *35S:DcMYB113* and *Pro_PPHZ_:DcMYB113* transgenic KRD extracts had one major peak in the chromatogram, peak 3, identified as cyanidin 3‐xylosyl (sinapoylglucosyl) galactoside (Cy3XSGG). The other four peaks, 1, 2, 4 and 5, were identified as cyanidin 3‐xylosyl (glucosyl) galactoside (Cy3XGG), cyanidin 3‐xylosylgalactoside (Cy3XG), cyanidin 3‐xylosyl (feruloylglucosyl) galactoside and cyanidin 3‐xylosyl (coumaroylglucosyl) galactoside, respectively.

### Glycosylation and acylation of anthocyanins in carrot by DcUCGXT1 and DcSAT1

The reaction scheme for Cy3XSGG formation from cyanidin‐3‐galactoside (Cy3G) in carrot has been proposed previously (Glassgen *et al.*, 1998). Two glycosyltransferases, UDP‐xylose: cyanidin 3‐galactoside xylosyltransferase (UCGXT) and UDP‐glucose: cyanidin 3‐xylosylgalactoside glucosyltransferase (UCGXGT), and an acyltransferase, sinapoyl‐Glc: anthocyanin acyltransferase (SAT), are required for Cy3XSGG biosynthesis (Figure 5b). Three QTLs associated with variations in Cy3XSGG in carrots have been proposed previously (Cavagnaro *et al.*, 2014). Q1 is located in the same region as *DcMYB7*, a gene related to anthocyanin biosynthesis in carrots with a purple root and petiole (Iorizzo *et al.*, 2019). A *UCGXT* and a *SAT* gene*,* namely *DcUCGXT1* and *DcSAT1*, with the best BLAST match to *UGT79B1* and *SAT* (At2g23000) from *Arabidopsis* (Montefiori *et al.*, 2011), was found within the Q3 and Q2 locus, respectively (Xu *et al.*, 2019b). However, the glycosylation and acylation activity of these two genes were not determined. In this study, the glycosylation activity of recombinant DcUCGXT1 protein (rDcUCGXT1) was determined. The purified rDcUCGXT1 acted on Cy3G with UDP‐xylose as the sugar donor, generating a new product identified as Cy3XG (Figure 5c), while protein extracted from the control did not. The acylation activity of DcSAT1 was determined by overexpression in DPP. The DPP explants transformed with *35S:DcSAT1* and the pCAMBIA 1301 vector (as control) generated purple calli, which were subjected to HPLC‐MS analyses. As a result, Cy3XSGG was detected as the main anthocyanin in all three *35S:DcSAT1* DPP calli lines but not in the control calli (Figure 5d). These results suggest that DcSAT1 enhances Cy3XSGG production in carrot.

### Expression patterns of *DcUCGXT1* and *DcSAT1* in carrots

**Figure 6:**
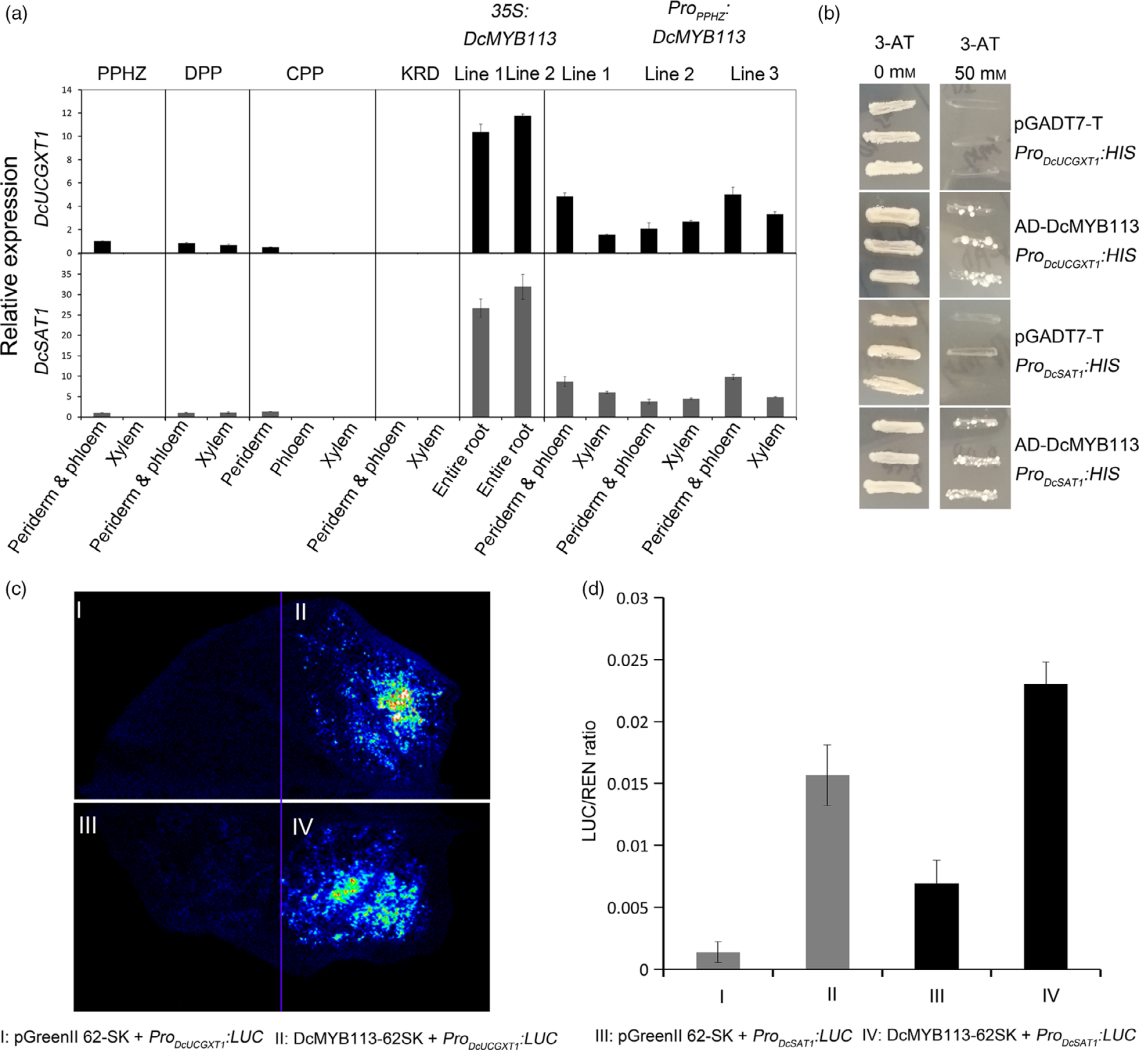
Regulation of *DcUCGXT1* and *DcSAT1* by DcMYB113*.* (a) Relative transcript levels of *DcUCGXT1* and *DcSAT1* in different root tissues of PPHZ, DPP, CPP, KRD, two *35S:DcMYB113* and three *Pro_PPHZ_:DcMYB113* transgenic KRD lines. Data are means of three biological replicates ± SDs. (b) Validation of interaction of DcMYB113 with the *DcUCGXT1* and *DcSAT1* promoter fragments by yeast one‐hybrid assays. (c) Transient expression analyses confirming transactivation of *DcUCGXT1* and *DcSAT1* by DcMYB113. (d) Transactivation activity of DcMYB113 on *DcUCGXT1* and *DcSAT1* expressed as ratio of luciferase (LUC) to Renilla (REN) activity. Data are means of six replicate reactions ± SDs.

Transcriptional analyses *via* qRT‐PCR indicated that *DcUCGXT1* and *DcSAT1* expression was associated with anthocyanin pigmentation. Both genes showed high transcript levels in all the purple root tissues of PPHZ, DPP and CPP, but were virtually undetectable in all the nonpurple root tissues of PPHZ, CPP and KRD (Figure 6a). *DcUCGXT1* and *DcSAT1* also showed sharply increased transcript levels in the *35S:DcMYB113* and *Pro_PPHZ_:DcMYB113* transgenic KRD lines compared with untransformed KRD, supporting the idea that *DcMYB113* regulated *DcUCGXT1* and *DcSAT1* in carrots.

### Activation of *DcUCGXT1* and* DcSAT1* expression by DcMYB113

Yeast one‐hybrid assays were conducted to determine whether DcMYB113 could bind to the promoter sequences of *DcUCGXT1* and *DcSAT1. Pro_DcUCGXT1_:HIS* or *Pro_DcSAT1_:HIS* was transformed into yeast cells with the pGADT7‐DcMYB113, generating transformants that were selected on SD/‐Trp/‐Leu/‐His plates supplemented with 50 mm 3‐amino‐1,2,4‐triazole (3‐AT) (Figure 6b). Yeast cells harbouring the control vectors could not survive on the selection medium. These results suggest that DcMYB113 binds to the promoter regions of *DcUCGXT1* and *DcSAT1*.

The dual‐luciferase (LUC) reporter system was used to verify the *DcMYB113‐*induced activation of the promoters of *DcUCGXT1* and *DcSAT1*. The DcMYB113‐62SK recombinant plasmid containing the CDS of DcMYB113 was inserted into the pGreenII 62‐SK vector. This recombinant vector was co‐infiltrated into *Nicotiana benthamiana* leaves with the construct *Pro_DcUCGXT1_:LUC* or *Pro_DcSAT1_:LUC* (containing the promoter sequence of *DcUCGXT1* or *DcSAT1,* respectively, fused to luciferase in pGreenII 0800 vector). In luminescence imaging assays, the tissues co‐expressing DcMYB113‐62SK and *Pro_DcUCGXT1_:LUC* or *Pro_DcSAT1_:LUC* showed strong luminescence intensity, whereas no visible or weak luminescence signals were detected in the control (Figure 6c). The transactivation activity of DcMYB113‐62SK on *Pro_DcUCGXT1_:LUC* was >11‐fold higher than that in the control, and on *Pro_DcSAT1_:LUC* was >3‐fold higher than that in the control (Figure 6d). Together, these results indicate that DcMYB113 activates the expression of *DcUCGXT1* and *DcSAT1* in carrot.

### Co‐expression of genes associated with DcMYB113 promoted anthocyanin biosynthesis in carrot root

**Figure 7:**
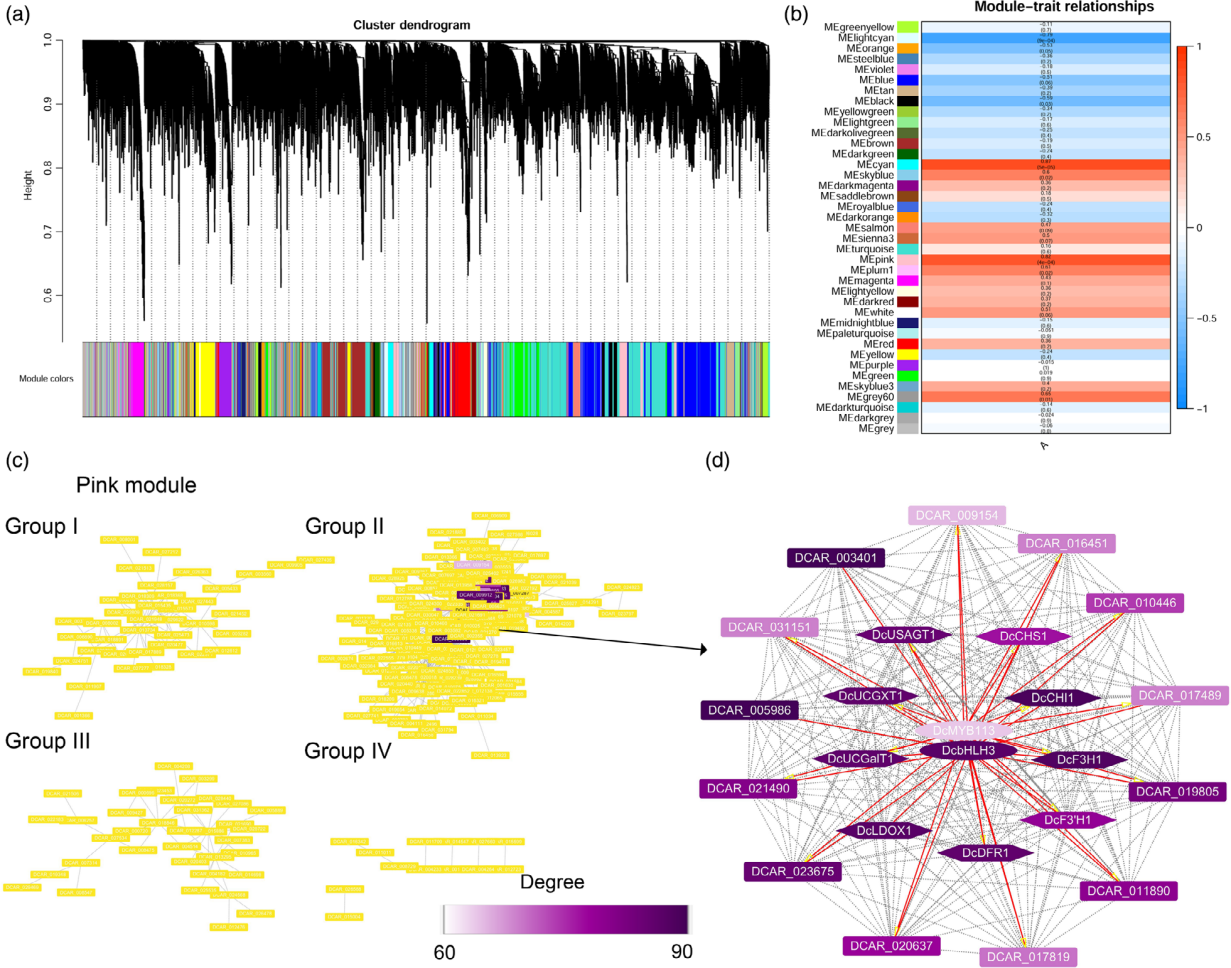
Weighted gene co‐expression network analysis of genes expressed in root tissues of PPHZ, CPP, KRD, as well as two *35S:DcMYB113* and three *Pro_PPHZ_:DcMYB113* transgenic KRD lines. (a) Hierarchical cluster tree showing 39 co‐expression modules with different colours in lower panel. Each gene is represented as a leaf in the tree. (b) Module‐trait (total anthocyanin content) correlations and *P*‐values (in parentheses). Colour in right panel shows correlation from –1 (blue) to 1 (red). In left panel, 39 modules are represented by different colours. (c) Genes identified in ‘pink’ module are clustered in four groups. Colour indicates node connectivity (degree) value lower than 60 (yellow), or from 60 (white) to 90 (purple). (d) Cytoscape representation of co‐expressed genes with edge weight ≥0.10 in ‘pink’ module. Colour shows node connectivity (degree) value from 60 (white) to 90 (purple).

A weighted gene co‐expression network analysis (WGCNA) was conducted using RNA‐Seq data and total anthocyanins contents of root tissues of PPHZ, KRD, CPP (excluding periderm tissues), as well as the two *35S:DcMYB113* and three *Pro_PPHZ_:DcMYB113* transgenic KRD lines. A total of 8847 genes were retained for the WGCNA after filtering, leading to the generation of 39 modules based on the pairwise correlations of gene expression across all samples (Figure 7a, Table S4). It is notable that *DcMYB113* was identified in ‘pink’ module. The module–trait relationships indicated that the ‘pink’ module was highly positively correlated with total anthocyanin contents (*r* = 0.82, *P* = 4 × 10^−4^) (Figure 7b).

A total of 311 genes with edge weight >0.10 were identified in the ‘pink’ module. Cytoscape representation indicated that the 311 genes were clustered into four groups (I, II, III and IV group) (Figure 7c). A total of 24 co‐expressed genes in group II showing a high node connectivity (degree > 60) were identified and regarded as Hub genes (Figure 7d, Table S5). *DcMYB113*, *DcbHLH3*, *DcCHS1*, *DcCHI1*, *DcF3H1*, *DcF3'H1*, *DcDFR1*, *DcLDOX1*, *DcUCGalT1*, *DcUCGXT1* and *DcUSAGT1* (Chen *et al.*, 2016) were identified in the 24 Hub genes. However, *DcSAT1* was not found in the Hub genes because there was no corresponding predicted gene in the carrot genome. DCAR_003401, a gene encoding glutathione S‐transferase (GST), and DCAR_031551, a gene encoding a multidrug and toxic compound extrusion (MATE) transporter, with the best BLAST match to *GST* (Gomez *et al.*, 2011) and *anthoMATE1* (Gomez *et al.*, 2009) that were responsible for anthocyanin transport in grapevine, respectively, were also identified. DCAR_003401 (*GST*) showed the highest node connectivity among the 24 Hub genes. Comparative transcriptome analyses of RNA‐Seq data revealed that DCAR_003401 (*GST*) and DCAR_031551 (*MATE*) expression was associated with DcMYB113 expression (Table S6).

Of the remainder 11 Hub genes, DCAR_021490 (*DcF5H*) is related to lignin biosynthesis (Wang *et al.*, 2016a); DCAR_019805 is homologous to *AtCHIL*, which promotes flavonoid production (Jiang *et al.*, 2015); DCAR_005986 is homologous to *AtCYP714A1*, which deactivates gibberellin (Zhang *et al.*, 2011); DCAR_010446 is homologous to *AtREF1*, which is involved in ferulic acid and sinapic acid biosynthesis (Nair *et al.*, 2004); DCAR_009154 is homologous to *AtTAF12*, which negatively regulates cytokinin sensitivity (Kubo *et al.*, 2011). In addition, DCAR_011890, DCAR_020637, DCAR_017819 and DCAR_016451 are homologous to *transmembrane amino acid transporter* (AT3G56200), *MATE transporter* (AT3G03620), *PATATIN‐like 4* (AT4G37050) and *PPR containing protein* (AT3G53170) from *Arabidopsis thaliana* with unknown function; DCAR_023675 and DCAR_017489 encode unknown protein. None of them has been reported to involve in anthocyanin biosynthesis or relate to other functions so far.

## Discussion

It is thought that purple and yellow carrots were the first domesticated carrots, based on historical documents (Banga, 1963). Modern domesticated purple carrot cultivars show various purple pigmentation types in roots, ranging from purple pigmentation only in the periderm to solid purple roots. The molecular and biochemical basis of genetic factors conditioning anthocyanin pigmentation in carrot roots remains unclear (Cavagnaro *et al.*, 2014; Simon, 1996; Xu *et al.*, 2014; Yildiz *et al.*, 2013). Two dominant genes, *P_1_* and *P_3_*, have been proposed to be responsible for anthocyanin pigmentation in purple carrot roots and have been genetically mapped to two different loci of chromosome 3 in previous reports (Cavagnaro *et al.*, 2014; Simon, 1996; Zhou *et al.*, 2015). Recently, *DcMYB7* gene was identified as a candidate for *P_3_* gene (Iorizzo *et al.*, 2019). *DcMYB7* expression is associated with purple pigmentation in carrot with purple root and petiole. However, the candidate for *P_1_*, a gene responsible for anthocyanin pigmentation in carrot with a purple root and nonpurple petiole, has not been identified yet. In the present study, we conducted comparative transcriptome and phylogenetic analyses for genes located in the *P_1_* region and identified a gene, *DcMYB113*, as a candidate gene for *P_1_*. *DcMYB113* specifically expressed in root of the carrot cultivar with a purple root and nonpurple petiole, PPHZ. *DcMYB113* transcripts were undetectable in purple or nonpurple root tissues of DPP, CPP and KRD. The promoter sequences of *DcMYB113* differed between PPHZ and the other three carrot cultivars, supporting the idea that changes in the promoters have led to the transcriptional inactivation of *DcMYB113* in the DPP, CPP and KRD roots.

The anthocyanin inducing function of *DcMYB113* from PPHZ was verified in CPP and KRD. Overexpression of *35S:DcMYB113* in KRD resulted in anthocyanin biosynthesis in both root and petiole, while *Pro_PPHZ_:DcMYB113* transgenic KRD lines had a purple root and nonpurple petiole. These results suggested that *DcMYB113* conditioning anthocyanin pigmentation specific in root was determined by its promoter. Unlike the roots with nonpurple (orange) xylem tissue in PPHZ and B7262, the inbred lines used for *P_1_* genetic mapping in a previous study (Cavagnaro *et al.*, 2014), *Pro_PPHZ_:DcMYB113* transgenic KRD roots had purple xylem tissue. In addition, *DcMYB113* expressed in root xylem tissue of *Pro_PPHZ_:DcMYB113* transgenic KRD lines but not in that of PPHZ, supporting the speculation that another genetic factor induced *DcMYB113* expression in root xylem tissue of *Pro_PPHZ_:DcMYB113* transgenic KRD or suppressed *DcMYB113* expression in the root xylem tissue of PPHZ. This genetic factor has never been reported before. Future studies should focus on investigating it.

In many plant species, MYBs interact with bHLHs and WD repeats to form a MBW complex that directly regulates anthocyanin accumulation. In fact, MYBs and bHLHs are both essential for anthocyanin accumulation in some plant species (Chagne *et al.*, 2013; Espley *et al.*, 2007). Here, *DcbHLH3* is positively regulated by *DcMYB113*. Yeast two‐hybrid assays showed that DcMYB113 can interact with the DcbHLH3 protein. All tested anthocyanin pathway structural genes also showed increased transcript levels in KRD carrying *35S:DcMYB113*. Therefore, we propose that, like in *Arabidopsis*, DcMYB113 up‐regulates its partner DcbHLH3 and together they induce anthocyanin biosynthesis in carrot by up‐regulating all the anthocyanin biosynthetic structural genes (Tohge *et al.*, 2005). This is in contrast to the situation in apple, where bHLH3 is not up‐regulated by MYB10 (Espley *et al.*, 2007). The transcript level of *DcbHLH3* was positively correlated with anthocyanin accumulation in all tested purple carrot cultivars, suggesting that *DcBHLH3* is involved in anthocyanin biosynthesis in all three tested types of purple carrot roots.

The structural genes, *CHS*, *CHI*, *F3H*, *F3'H*, *DFR*, *LDOX* and *UFGT*, are responsible for anthocyanin biosynthesis in purple carrots, like in many other plant species. Anthocyanins undergo further modifications, such as glycosylation, acylation and methylation in plants. The attachment of different moieties significantly affects the stability and bioavailability of anthocyanins. In purple carrots, anthocyanins are further glycosylated and acylated by glycosyltransferases and acyltransferases, respectively. Cavagnaro et al. found several QTLs associated with variations in anthocyanin glycosylation and acylation, but did not identify any candidate genes (Cavagnaro *et al.*, 2014). Following on from that important work, *DcUCGXT1* and *DcSAT1*, encoding a glycosyltransferase and an acyltransferase, respectively, were found within two QTLs (Xu *et al.*, 2019b). In this study, we confirmed that *DcUCGXT1* catalysed the synthesis of Cy3XG by adding xylose to Cy3G and *DcSAT1* catalysed the addition of sinapoylglucose to anthocyanins. Considering that both *DcUCGXT1* and *DcSAT1* were highly expressed in *35S:DcMYB113* and *Pro_PPHZ_:DcMYB113* KRD lines but not in the untransformed ones, it was reasonable to speculate that *DcMYB113* bound to the promoters of *DcUCGXT1* and *DcSAT1* and activated their expression. These interactions were confirmed through yeast one‐hybrid and dual‐luciferase reporter assays in this study. These data suggest that *DcMYB113* mediates routes from Cy3G to the Cy3XSGG in the intricate anthocyanin modification pathways in carrot.

Using WGCNA, we identified a module containing the co‐expression gene network with 24 Hub genes viewed to associate with anthocyanin accumulation in carrot. *DcMYB113*, *DcbHLH3* and several structural genes responsible for anthocyanin biosynthesis and modification were identified within the Hub genes, showing the power of WGCNA in the identification of a co‐expression gene network involved in anthocyanin accumulation in carrot. The WGCNA results showed that *DcMYB113* co‐expressed with genes homologous to the *GST* and *MATE*, which were responsible for anthocyanin transport (Gomez *et al.*, 2011; Gomez *et al.*, 2009), suggesting that *DcMYB113* may condition anthocyanin transport in carrot. In addition, homologs of genes previously known to have roles in flavonoid accumulation, gibberellin deactivation, lignin biosynthesis, as well as ferulic acid and sinapic acid production were found within the 24 Hub genes, implying *DcMYB113* may be also involved. Moreover, the remainder 6 Hub genes with unknown function provided potential gene resources co‐expressed with anthocyanin production in carrots. This knowledge of co‐expression gene network provided a comprehensive level view in anthocyanin pathway.

## Conclusion

An *R2R3‐MYB*, *DcMYB113*, was identified within the *P_1_* locus on chromosome 3 of carrot. *DcMYB113* expression was associated with root‐specific anthocyanin pigmentation in PPHZ, a carrot cultivar with a purple root and nonpurple petiole, but not in DPP, CPP and KRD. Transcriptional inactivation of *DcMYB113* in the DPP, CPP and KRD roots appears to be caused by variation in the promoters region. *DcMYB113* from PPHZ complemented CPP and KRD anthocyanin‐deficient phenotype. An unknown genetic factor conditioning anthocyanin pigmentation in root xylem tissue by regulating *DcMYB113* expression was proposed based on the different anthocyanin distribution in roots of PPHZ and *Pro_PPHZ_:DcMYB113* transgenic KRD lines. *DcMYB113* activated the expression of its bHLH partner and all the tested anthocyanin biosynthetic structural genes. *DcUCGXT1* and *DcSAT1*, which were responsible for anthocyanins glycosylation and acylation, respectively, were also activated by *DcMYB113*. The WGCNA results indicated *DcMYB113* may regulate anthocyanin transport and identified several genes co‐expressed with anthocyanin biosynthesis. These results advanced our knowledge on the genetic control of root‐specific anthocyanin pigmentation and provided the molecular mechanism underlying anthocyanin modifications in carrot. Our findings will also be useful for further research on, and manipulation of, anthocyanin composition in carrots and other root crops.

## Materials and methods

### Physical map anchoring

The sequences of markers (SNPs and SSRs) are provided in three previous reports (Baranski *et al.*, 2012; Cavagnaro *et al.*, 2014; Iorizzo *et al.*, 2013). These markers were anchored to a physical map with Mapchart version 2.32 (https://www.wur.nl/en/show/MapChart-2.32.htm.).

### Multiple sequence alignment and phylogenetic analysis of R2R3‐MYB transcription factors

The predicted protein sequences of DcMYB113 were downloaded from a doubled haploid carrot genome sequencing project (Iorizzo *et al.*, 2016). The protein sequences of 125 *A. thaliana* R2R3‐MYB TFs were obtained from TAIR (http://www.arabidopsis.org/). The full‐length sequences of R2R3‐type MYB TFs were subjected to a multiple alignment analysis with ClustalW (http://www.ebi.ac.uk/Tools/msa/clustalw2/). Phylogenetic analyses were conducted with MEGA5 (http://www.megasoftware.net/) using the neighbour‐joining (NJ) method with 1000 bootstrap replicates.

### Plant materials and growth conditions

Carrots were grown in an artificial climate chamber under the same conditions as previously described (Xu *et al.*, 2014). PPHZ is a carrot cultivar with purple root periderm and phloem tissues but orange root xylem tissue and a nonpurple petiole. DPP is a carrot cultivar with a solid purple root and purple petiole. CPP is a carrot cultivar with a purple root periderm tissue and petiole but orange root phloem and xylem tissues. KRD is a carrot cultivar with an orange root and nonpurple petiole. Young leaves of carrot plants were used for genomic DNA extraction. Anthocyanins and total RNA were extracted from various root tissues of 90‐day‐old carrot plants. Periderm, phloem and xylem tissues were separately collected from CPP roots as samples. Because successful discrimination and excision of periderm tissue from the phloem tissue were impossible for roots of KRD, PPHZ and DPP, these two tissues were collected together as ‘periderm & phloem’ sample. After harvest, these samples were frozen in liquid nitrogen and then stored at −80°C until use.

### Anthocyanin extraction and measurement

Total anthocyanins were extracted from different carrots with extraction buffer (49.9% ddH_2_O, v/v; 0.1% HCl, v/v; and 50% methanol, v/v). The anthocyanin content and composition profile were determined as described elsewhere (Feng *et al.*, 2018; Li *et al.*, 2012).

### *De novo* genome assembly

Raw reads data (GenBank accession number, SRR2146943) of a purple carrot cultivar (GenBank accession number, SAMN03766331) with a purple root and nonpurple petiole were downloaded from the Sequence Read Archive (SRA). SOAP denovo version 2.04 was used for genome assembly.

### Genomic DNA, total RNA extraction, gene cloning and qRT‐PCR assays

The genomic DNA and total RNA isolation, first‐strand cDNA synthesis, gene amplification and qRT‐PCR assays were performed using the same methods as described previously (Wang *et al.*, 2015; Xu *et al.*, 2017). Experiments were conducted in biological triplicate for each sample. The relative transcript levels of gene were normalized to that of *DcActin1* and calculated with the 2^−ΔΔCT^ method (Schmittgen and Livak, 2008; Wang *et al.*, 2016b). All the primers used in this study are listed in Table S3 or in our previous report (Xu *et al.*, 2014).

### Generation of transgenic carrots

*DcMYB113* CDS was amplified from the cDNA library of PPHZ and was introduced into the pCAMBIA 1301 vector under the control of the CaMV 35S promoter to generate the *35S:DcMYB113* construct. The 2,388‐bp promoter and CDS region of *DcMYB113* were amplified from PPHZ and used to create the *Pro_PPHZ_:DcMYB113* construct. The recombined vectors were genetically transformed into carrot by *A. tumefaciens* (GV3101)‐mediated transformation as described previously (Xu *et al.*, 2019a). The primers used are listed in Table S3.

### Yeast two‐hybrid assay

The CDSs of *DcMYB113* and *DcbHLH3* were cloned from PPHZ and *35S:DcMYB113* KRD, respectively. Then, they were separately inserted into the pGADT7 and pGBKT7 vectors (Clontech, Palo Alto, CA, USA) to generate the AD‐DcMYB113 and BD‐DcbHLH3 constructs, respectively. These constructs were co‐transformed into cells of the yeast strain Y2HGold (Clontech) with pGADT7 and pGBKT7 vectors used as the negative controls. After incubation on SD/–Leu/–Trp plates at 30°C for 3–4 days, the transformants were transferred onto SD/‐Ade/‐His/‐Leu/‐Trp plates with or without X‐α‐Gal to detect interactions.

### Expression of DcUCGXT1 in *E. coli* and activity assays

The CDS of *DcUCGXT1* was amplified from the cDNA library of *35S:DcMYB113* KRD and cloned into the pET‐30a vector (Novagen, Madison, WI, USA) between the *Bam* HI and *Hind* III sites. This construct or the pET‐30a vector was then transformed into *E. coli* BL21 (DE3), and expression was induced in LB medium containing 1.0 mm isopropyl β‐D‐1‐thiogalactopyranoside (IPTG) for over 12 h at 18°C. rDcUCGXT1 was purified as described previously (Xu *et al.*, 2016). Crude protein was extracted from *E. coli* transformed with the pET‐30a vector as the control. Enzyme activity was detected in 50 mm NaH_2_PO_4_‐Na_2_HPO_4_ (pH = 7.5) buffer containing 0.4 mm UDP‐xylose (CarboSource Services, University of Georgia, Athens, GA, USA), 0.2 mm cyanidin‐3‐*O*‐galactoside (Sigma‐Aldrich, St Louis, MO, USA) and about 5 μg protein in a final volume of 50 μL. The mixture was incubated at 30°C for 1 h, and the reaction was terminated by adding of 2 μL 12 m HCl. The reaction products were analysed by HPLC‐MS.

### Functional analysis of *DcSAT1*

The CDS of *DcSAT1* was amplified from *35S:DcMYB113* transgenic KRD lines and cloned into the pCAMBIA 1301 vector under the control of the 35S promoter to create the *35S:DcSAT1* construct. The recombined vectors and pCAMBIA 1301 (as control) were genetically transformed into DPP. The anthocyanin composition profile in calli generated from explants was analysed by HPLC‐MS.

### Yeast one‐hybrid assay

The promoter of *DcUCGXT1* or *DcSAT1* was amplified from KRD genomic DNA and fused to histidine (HIS) in the pHis2 vector to generate *Pro_DcUCGXT1_:HIS* or *Pro_DcSAT1_:HIS*, respectively. These two constructs were separately co‐transformed with AD‐DcMYB113 or the pGADT7‐T vector (control) into cells of the Y1H Gold yeast strain (Clontech). The interactions were tested on the same medium as described in a previous report (An *et al.*, 2018), but containing 50 mm 3‐AT. The primers used in this procedure are listed in Table S3.

### Transient expression and dual‐luciferase reporter assays

*DcMYB113* from PPHZ was subcloned into the pGreenII 62‐SK vector to prepare the DcMYB113‐62SK effector construct. The promoter fragments of *DcUCGXT1* and *DcSAT1* were separately introduced into the pGreenII 0800‐LUC vector to create the reporter constructs. The effector and reporter constructs were co‐introduced into *N. benthamiana* leaves for transient expression using *A. tumefaciens* (GV3101)‐mediated transformation. The luminescence of firefly luciferase was detected as described previously (Li *et al.*, 2017). The ratio of luminescence of firefly luciferase to Renilla luciferase was detected as described elsewhere (Chagne *et al.*, 2013). The primers used in this procedure are listed in Table S3.

### RNA sequencing data analysis and weighted gene co‐expression network analysis

RNA sequencing was conducted using the Illumina HiSeq platform with cDNA libraries prepared from various root tissues of PPHZ, CPP, KRD, DPP, *35S:DcMYB113* and *Pro_PPHZ_:DcMYB113* transgenic KRD lines. After quality control and filtering, clean RNA‐Seq reads were mapped to the carrot genome (V2.0) with gene annotation using Bowtie v2.1.0 (Langmead and Salzberg, 2012). Gene expression levels were calculated and normalized by FPKM values. A WGCNA was conducted to identify genes related to anthocyanin biosynthesis using the R package (Langfelder and Horvath, 2008). Network construction and module detection were performed as described previously (El‐Sharkawy *et al.*, 2015). The network of modules of co‐expressed genes (edge weight >0.10) was represented by Cytoscape 3.7.1 (Saito *et al.*, 2012).

## Accession numbers

The RNA‐Seq raw reads of root tissues of PPHZ, DPP, CPP and KRD have been deposited in the Sequence Read Archive (SRA) under project PRJNA540112, accession SRP194046. The RNA‐Seq raw reads of root tissues of *35S:DcMYB113* and *Pro_PPHZ_:DcMYB113* transgenic KRD lines have been deposited in the SRA under project PRJNA548023, accession SRP200826. The gDNA, cDNA and promoter sequences of *DcMYB113* from PPHZ have been deposited at the GenBank database under the accession numbers MK896874, MK896875 and MN149519, respectively.

## Author contributions

Z. S. X. and A. S. X. initiated and designed the research. Z. S. X., Q. Q. Y. and X. Y. performed the experiments. Z. S. X. and K. F. analysed the data. Z. S. X. and A. S. X. contributed reagents/materials/analysis tools. Z. S. X. wrote the paper. Z. S. X. and A. S. X. revised the paper.

## Conflict of interest

All co‐authors declared that they have no conflicts of interest in this work.

## Supporting information


**Figure S1** Phylogenetic tree of DcMYB113 (DCAR_008994) and R2R3‐MYB TFs from A. thaliana.
**Figure S2** The contig containing DcMYB113 from the assembled RNA‐Seq reads of PPHZ roots. The coding sequence of DcMYB113 is marked in red.
**Figure S3** Alignment analysis of the DNA sequence and coding sequence of DcMYB113 from ‘Purple haze’. Identical sequences are shaded with black. The threshold for shading was set to 60%.
**Figure S4** The scaffold containing promoter and partial DNA sequences (red mark) of DcMYB113 from the assembled reads (Accession number, SRR2146943).

**Table S1** A total of 748 genes identified within the P1 region based on the RNA‐Seqs of PPHZ, DPP, CPP, and KRD.
**Table S2** The eleven genes showing greater than 5‐fold fragments per kilobase per total million mapped reads (FPKM) increase in the RNA‐Seqs of purple root periderm and phloem tissues of PPHZ than that of other root tissues of PPHZ, DPP, CPP, and KRD. The genes with expression level greater than 2 FPKM in the root periderm and phloem tissues of PPHZ are marked in red.
**Table S3** Primers used in this study.
**Table S4** The genes used for WGCNA.
**Table S5** The genes in group II of ‘pink’ module with a high node connectivity (degree >60).
**Table S6** The expression level of DcMYB113, DCAR_003401, and DCAR_031151 in different carrot root tissues according to transcriptome data.
