# Supplementary material for: DcMYB113, a root‐specific R2R3‐MYB, conditions anthocyanin biosynthesis and modification in carrot
Source: Plant Biotechnol J. 2020 Jan 22;18(7):1585–97. doi: 10.1111/pbi.13325 (PMC7292547; doi:10.1111/pbi.13325)
Supplement: Supplementary file 1 — Figure S1 Phylogenetic tree of DcMYB113 (DCAR_008994) and R2R3‐MYB TFs from A. thaliana. Figure S2 The contig containing DcMYB113 from the assembled RNA‐Seq reads of PPHZ roots. The coding sequence of DcMYB113 is marked in red. Figure S3 Alignment analysis of the DNA sequence and coding sequence of DcMYB113 from ‘Purple haze’. Identical sequences are shaded with black. The threshold for shading was set to 60%. Figure S4 The scaffold containing promoter and partial DNA sequences (red mark) of DcMYB113 from the assembled reads (Accession number, SRR2146943). [file PBI-18-1585-s002.doc]

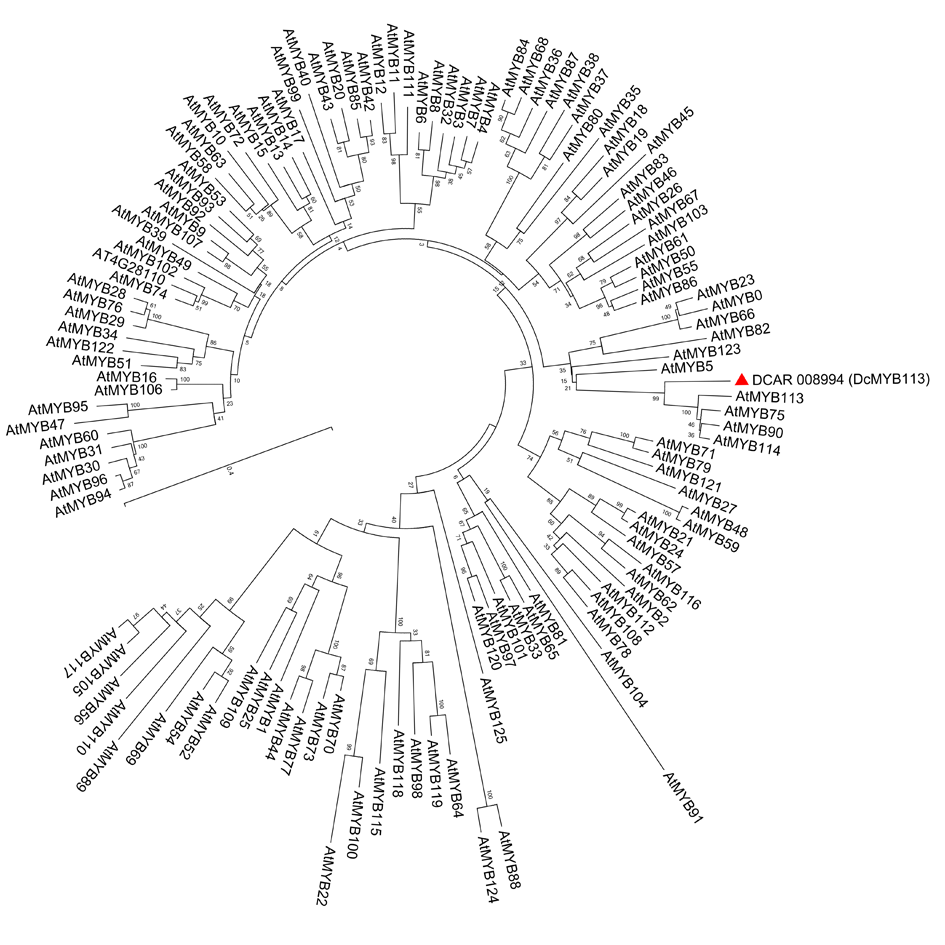


Figure S1. Phylogenetic tree of DcMYB113 (DCAR_008994) and R2R3-MYB TFs from *A. thaliana*.

CTTATGGTCTGCGTGTCCTTCAGTAATATCACATAAGGTTGCTACCTAGAACTTGCCTGAATGTCAGATTCTTGTCCTAGCTTGGTCCATTATGATTAGCTCAAATCTGAAAATGACCAATATTCACGTCATATTCTAACCGAGTCATAACTTTGATGTCACGTTGAGAACCAGTTCAAATGGATCTTATGTTACATTTCAACAATTTTATACCCAATAACTTCCACAAATTAAGGAATGAGGTACAGAAAATGACAGACAATTAATAAAAATCACAAGTAATCCATGGTAATTACTTGGGGAGGAAGGAAGCACGTCGAACCTTTGAAGAACTAAGATTCACCAACTCATTATATGTTGACAACAACACACCACAGTAATATTTATTATGGTGCAGCTTTAAGGGCCGTCTAGCTAGTATAAATAGAAGTTCACTTGGTTAGTATTCAAGTGCTTAATTATCCTCCAGTACCACTGCATCTATGTATAGTGTGGTAGTAATGAGCTATCCCAAGGCCATGAAGAGTGCTAACGCAGCTTCAAAGCTGCGAAAGGGTGCATGGGATTTTGAGGAAGACACTCTTCTCCGAAAATGCATTGACAAGTATGGAGAGGGAAAGTGGCACCTTGTTCCGCAGAGAGCTGGGTTGAATAGATGCAGAAAAAGTTGTAGGCTAAGGTGGCTTAACTATCTTAGGCCTACCATCAAGAGAGGAGAGTTCAGTGAGGATGAAGTGGATCTTATGATACGCCTTCACAGGCTGCTGGGAAACAGATGGTCGTTAATTGGGGGGAGACTGCCGGGAAGAACAGCTAATGATGTTAAGAACTATTGGAACACCAACATTCAAAAGAAGCTCGCTACTGGCAGCAACCAGAAAGAGATGGCAATAAAAGAAGAGTTCGTTCAAGGAAAACAAGATAGTAACATTGCTGCAACTAGTGGTGGTGGTTGTGCTGCTACTACTACTATTATTAAGCCTTTTCCCCGGATGTTATCCAAAGGCACAAGCCTACCCTGCTATAAACTTAATCTCAAGAATCGCGTGAGTTTTGGTTTAAATGACGATACACTACAGAATAACAACAATGAAAATAACAAGAAGCCATCATTGCAAGCAATGCTGCCATTGAATGACGAAAGCAATGAGACCCTGACACCGGATGAAGATGGCATAGAGTGGTGGAAGAATTTGTTTGCAGAAATAGATATTGATGGTCAAGAGCAAGATTCATCACAAGGACTATTGATGGCATCCTCGAGTGGTTTAGAAAATGCGGATGCAGACAGAGACCTAATGTGGAAAACTGATGAATCAACTGCTGCGGTAATGGAATTTTCGGATGACTTAAGTGGCATTTGGGATCTTCTAGACTCACCAGATTATGTCCGATTGAGTCGATTATAATATATAAAAATCCAGCAAATATCCTGTTGGTTTTATTAAGTTAAAGGTTTGAAATAATGATGTATTTTAATGATCTTGCTTGTTTAATGTTTATGTATGTACTGCTATTATTCTGCTTGGCATTGAACTTTGTTGTTCTTTGGCTTTGCAAGTATATTTTCTTTAATATACACTGTAATAATTCTTATTTATTATTCAAGATTTGTTGATGGGA

Figure S2. The contig containing *DcMYB113* from the assembled RNA-Seq reads of PPHZ roots. The coding sequence of *DcMYB113* is marked in red.


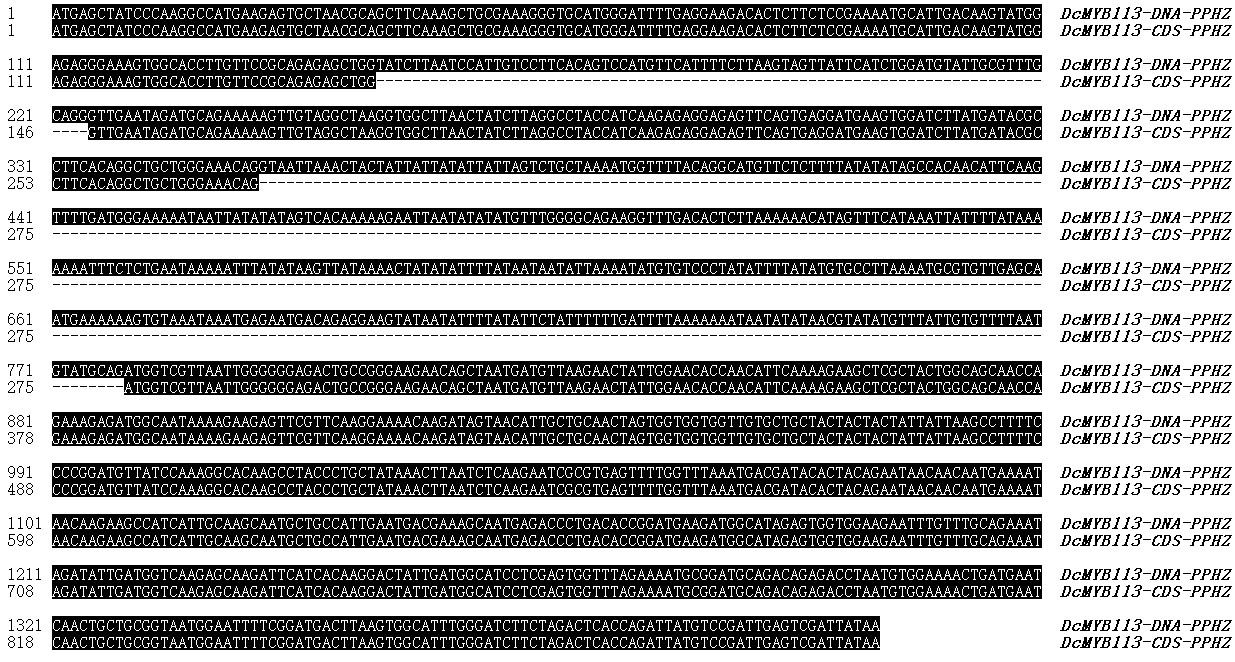


Figure S3. Alignment analysis of the DNA sequence and coding sequence of *DcMYB113* from ‘Purple haze’. Identical sequences are shaded with black. The threshold for shading was set to 60%.

GTTTAAAAATTGTTGTGCTGTGAAGAAAAGATATTGGTGAAAAAAAGTACAGTTGTAGAAATCTGATAACTACCTGATTTTTTTTTTTGATTTTTACNTTTTGATTGTAAAATCTGTAACTGATCCGGTAAAAACTTAAAAATAATTTTTTCAAAAAANNNNNNNNNNNNNNNNNNNNNNNNNNNNNNNNNNNNNNNNNNNNNNNNNNNNNNNNNNNNNNNNNNNNNNNNNNNNNNNAAAATTCTCGCGCTGACACCGAACACCAAACAGTACCTAACAACCCCGGTCTTTATTGTGTTTTAAACTTTTAATGTACCTATAGATAATCATGTCAGGACATTTTTGACTTGTTCATTTATCCAACTCAAATTTACAAATCTCTTTAAAACTGGAGATACGTGAATTCTACACATTAATACTTCATCATAATCTGAATTCTACATAATGGAATATTCCATCAGAATTTGATCCATGTTTCGCAAATGTACGCTGCGACTTTAAAGTCAGGGTAAAACAAAATAATGTTCAAGATAGGAACTATGATGAAATACAAACCAAAAAATGCGTGATGAAGTTGTCAGTTTTCGTAAAAGTACAGGATATTTCTGTACAAATAAAGTTCATCTTTTTTATTTAATAAAATTCTCAAAATTTGTTTTATATTAGAAAAGGAAATAAATAACTTGTAAATTTAATATCTAAAGTCAAAAAAATGGTATAAGAAGACGTAAAATATATATATTAAAATTAAAANNNNNNNNNNNNNNNNNNNNNNNNNNNNNNNNNNNNNNNNNNNNNNNNNNNNNNNNNNNNNNNNNNNNATATTATGGATAGTACAATATGTGATATAACGTTAAATTGACGGAAAATTGTACAATCGACTAATAATTCAGTGAAAATGCTATAGCATCTAGATTAATTTCAAAGAGGATTCGAGTCATAATTTAATTGATCACAATCTTATTATGTATATACAGCAATCATATCAATTAAAATATTTAAACATATCATCAACACTGTGTTATTTATAATTAAACAGGCATGAAAGATTCCGGTTGTCTTTTTCAATTTCGAAGATAGTTATAGTGATTAGTGACCCAACAAAATTGTGCGGAAGAGAGGGAGGGGCTCGCGGTCCCCCGGGGTCAGGGACCAGGAGCAAGATTGCTCAGCTTCTATATCAACATACTAAATTTTATACACATACTCATATATTGTTTTCTCCTTTATCATTGTACCATAATACTGTACGGCAAGAGCATCTTCAATGATGATGCTAAATCATTGCTTATAGATAATTGAGTATTTCCAACAGGTTTGGTTATAATTGTTGGTTAANNNNNNNNNNNNNNNNNNNNNNNNNNNNNNNNNNNNNNNNNNNNNNNNNNNNNNNNNNNNNNNTTAAGACTTGTTGAATTGTTCTTCAATGATATTGGTTATATNNNNNNNNNNNNNNNNNNNNNNNNNNNNNNNNNNNNNNNNNNNNNNNNNNNNNNNNNNNNNNNNNNNNNNNNNNNNNNNNNNNNNNNNNNNNNNNNNNNNNNNNNNNNNNNNNNNNNNNNNNNNNNNNNNNNNNNNNNGCATTAATTATATGTTTTTGTATTATTAATTATTCTTATATTAAACATTAATTAATATTTAATTACTTACATAGAGACAAATGATCCAAAATGAAATTGATTTTCATNNNNNNNNNNNNNNNNNNNNNNNNNNNNNNNNNNNNNATTCCATCTCATACCCATCTAGGGATGGTAAAAAAATCCGATTCGACAGATACCGATCCGAAAATAATTTGGATGTACCAAATCCGATTTTTTGGATATGGATTNNNNNNNNNNNNNNNNNNNNNNNNNNNNNNNNNNNNNNNNNNNNNNNNNNNNNNNNNNNNNNNNNNNNNNNNNNNNNNNNNNNNNNNNNNNNNNNNNNNNNNNNNTAGGTAATACCGACCCGAAACCTGAAATTCGATCCAAACCCGATTNNNNNNNNNNNNNNNNNNNNNNNNNNNNNTCGAAAAATACCCGAATACAAACAAACAACACTAACTAGGATGTAACTTCGATCGGTCCTTGTGTCTTCATAACCAAACGCCATCGCCCTCGTCATCACCAACATGAGTTTATCAATCAGTCTCGTGAAAACGGAAAATATGACAAATCGACAACAGTACTATTTTGTTTGTGTCTTACCAAGAAACAAGTGAGAGATCGAGATAAAAAAGATATCAAAATAACTGAGCCTGAGAGATCATTTGTTTTACTTTCTAATGAAGCTAAATGAAAATTATATTAACTGATCAACTACAGTTTGACAACTGTTTTTAACTTGGTGAATGTTTCATCACAGCCCCATTGGTCAGCTAGATGATGATAAGGGAGCGTTCAGGCATCACATTTCAACGAGCATATGCCTTCCCAATTCCCATAACTTTCTCTGATCCACAAGCGCATCACCATCACCATCATGAGTTGATCGATCAGTCCGGTGAAAACGAAAGATAACAAATTGATAGTAGTACTGTTTTGTGTGCCTATCACTGGCTAATTGCGGTACTAGTTACTTTTATATATGAGTACGAAGGATAACAATGCAAAGGTGCTCTTATNNNNNNNNNNNNNNNNNNNNNNNNNNNNNNNNGTCTGCGTGTCCTTCAGTAATATCACATAAGGTTGCTACCTAGAACTTGCCTGAATGTCAAATTCTCTCAAGATCTAATGTCCTAGCTAGCTTGGTCCATTATGATTAGCCCAAATCTGAAAATAACCAATTAATCACGTCATATTCTAAACAAGTTAATTAGAACTCTGATATCACGTTGAGAACNNNNNNNNNNNNNNNNNNNNNNNNNNNNNNNNNNNNNNNNNNNNCTTCCACAAATTAAGGAATGAGGTACAGAAAACGACAAACAATTAATAAAAATCACAAGTAACTCATGGTAATTACTTGGGTAGGAAGGAAGCACGTCGAACCTTTGAAGAACTAAGATTCACAAACTCATTATATGTTGACAACAACACACTACAGTATTTATTATGGTGCAGCTTTAAGGGCCGTCTAGCTAGTATAAATAGAAGTTCACTTGGTTAGTATTCAAGTGCTTAATTATCCTCCAGTACCACTGCATCTATGTATAGTGTGGTAGTAATGAGCTATCCCAAGGCCATGAAGAGTGCTAACACAGCTTCAAAGCTGCGAAAGGGTNNNNNNNNNNNNNNNNNNNNNNNNNNNNNNNNNNNNNNNNNNNNGCAGAGAGCTGGTATCTTAATCCATTGTCCTTTAGTACAGTCCATGTTCATTTTCTTAAGGAGTAGTGCTAGGTGCAC

Figure S4. The scaffold containing promoter and partial DNA sequences (red mark) of *DcMYB113* from the assembled reads (Accession number, SRR2146943).
